# Tsallis Entropy of a Used Reliability System at the System Level

**DOI:** 10.3390/e25040550

**Published:** 2023-03-23

**Authors:** Mohamed Kayid, Mashael A. Alshehri

**Affiliations:** 1 Department of Statistics and Operations Research, College of Science, King Saud University, P.O. Box 2455, Riyadh 11451, Saudi Arabia; 2 Department of Quantitative Analysis, College of Business Administration, King Saud University, Riyadh 11362, Saudi Arabia

**Keywords:** coherent system, residual Tsallis entropy, Shannon entropy, system signature

## Abstract

Measuring the uncertainty of the lifetime of technical systems has become increasingly important in recent years. This criterion is useful to measure the predictability of a system over its lifetime. In this paper, we assume a coherent system consisting of *n* components and having a property where at time t, all components of the system are alive. We then apply the system signature to determine and use the Tsallis entropy of the remaining lifetime of a coherent system. It is a useful criterion for measuring the predictability of the lifetime of a system. Various results, such as bounds and order properties for the said entropy, are investigated. The results of this work can be used to compare the predictability of the remaining lifetime between two coherent systems with known signatures.

## 1. Introduction

For engineers, the performance and quantification of uncertainties over the lifetime of a system is critical. The reliability of a system decreases as uncertainty increases, and systems with longer lifetimes and lower uncertainty are better systems (see, e.g., Ebrahimi and Pellery, [1]). It has found applications in numerous areas described in Shannon’s seminal work, [2]. Information theory provides a measure of the uncertainty associated with a random phenomenon. If *X* is a nonnegative random variable with an absolutely continuous cumulative distribution function (CDF) F(x) and density function f(x), the Tsallis entropy of order α, defined by (see [3]), is

(1) $$\begin{array}{ccc} H_{\alpha }\left(X\right) & = & H_{\alpha }\left(f\right)=\frac{1}{1-\alpha }\left[\int_{0}^{\infty }f^{\alpha }\left(x\right)dx-1\right],\\ & = & \frac{1}{1-\alpha }\left[E\left(f^{\alpha -1}\left(X\right)\right)-1\right] \end{array}$$

for all α>0,α≠1, where E(·) denotes the expected value. In general, the Tsallis entropy can be negative, but it can also be non-negative if one chooses an appropriate value for α. It is obvious that $H\left(f\right)=\lim_{\alpha \to 1}H_{\alpha }\left(f\right)$ and thus reduces to the Shannon differential entropy. It is known that the Shannon differential entropy is additive in the sense that for two independent random variables *X* and *Y*, H(X,Y)=H(X)+H(Y), where (X,Y) denotes the common random variable. However, the Tsallis entropy is non-additive in the sense that $H_{\alpha }\left(X,Y\right)=H_{\alpha }\left(X\right)+H_{\alpha }\left(Y\right)+\left(1-\alpha \right)H_{\alpha }\left(X\right)H_{\alpha }\left(Y\right).$ Because of the flexibility of Tsallis entropy compared to Shannon entropy, non-additive entropy measures find their justification in many areas of information theory, physics, chemistry, and engineering.

If *X* denotes the lifetime of a new system, then $H_{\alpha }\left(X\right)$ measures the uncertainty of the new system. In some cases, agents know something about the current age of the system. For example, one may know that the system is in operation at time *t* and is interested in measuring the uncertainty of its remaining lifetime, that is, $X_{t}=X-t|X>t$. Then $H_{\alpha }\left(X\right)$ is no longer useful in such situations. Accordingly, the residual Tsallis entropy is defined as

(2) $$\begin{array}{ccc} H_{\alpha }\left(X_{t}\right) & = & \frac{1}{1-\alpha }\left[\int_{0}^{\infty }f_{t}^{\alpha }\left(x\right)dx-1\right]=\frac{1}{1-\alpha }\left[\int_{t}^{\infty }{\left(\frac{f(x)}{S(t)}\right)}^{\alpha }dx-1\right], \end{array}$$

(3) $$\begin{array}{ccc} & = & \frac{1}{1-\alpha }\left[\int_{0}^{1}f_{t}^{\alpha -1}\left(S_{t}^{-1}\left(u\right)\right)du-1\right],\;\alpha >0, \end{array}$$

where

$$f_{t}\left(x\right)=\frac{f(x+t)}{S(t)},\;x,t>0,$$

is the probability density function (PDF) of $X_{t},$S(t)=P(X>t) is the survival function of *X* and $S_{t}^{-1}\left(u\right)=\inf\left\{x;S_{t}\left(x\right)\geq u\right\}$ is the quantile function of $S_{t}\left(x\right)=S\left(x+t\right)/S\left(t\right),x,t>0$. Various properties, generalizations and applications of $H_{\alpha }\left(X_{t}\right)$ are investigated by Asadi et al. [4], Nanda and Paul [5], Zhang [6], Irshad et al. [7], Rajesh and Sunoj [8], Toomaj and Agh Atabay [9], Mohamed et al. [10], among others.

Several properties and statistical applications of Tsallis entropy have been studied in the literature, which you can read in Maasoumi [11], Abe [12], Asadi et al. [13] and the references therein. Recently, Alomani and Kayid [14] investigated some additional properties of Tsallis entropy, including its connection with the usual stochastic order, as well as some other properties of the dynamical version of this measure and bounds. Moreover, they investigated some properties of Tsallis entropy for the lifetime of a coherent and mixed system. It is suitable to study the behavior of the uncertainty of the new system in terms of Tsallis entropy. For other applications and researchers concerned with measuring the uncertainty of reliability systems, we refer readers to [15,16,17,18] and the references therein. In contrast to the work of Alomani and Kayid [14], the aim of this work is to study some uncertainty properties of a coherent system consisting of *n* components and having the property that at time t, all components of the system are alive. In fact, we generalize the results of the work published in the literature. To this end, we use the concept of system signature to determine the Tsallis entropy of the remaining lifetime of a coherent system.

The results of this paper are organized as follows: In Section 2, we provide an expression for the Tsallis entropy of a coherent system under the assumption that all components have survived to time *t*. For this purpose, we used the concept of system signature when the lifetimes of the components in a coherent system are independent and identically distributed. The ordering properties of the residual Tsallis entropy of two coherent systems are studied in Section 3 based on some ordering properties of system signatures even without simple calculations. Section 4 presents some useful bounds. Finally, Section 5 gives some conclusions and further detailed remarks.

Throughout the paper, “$\leq_{st}$”, “$\leq_{hr}$”, “$\leq_{lr}$” and “$\leq_{d}$” stand for stochastic, hazard rate, likelihood ratio and dispersive orders, respectively; for more details on these orderings, we refer the reader to Shaked and Shanthikumar [19].

## 2. Tsallis Entropy of the System in Terms of Signature Vectors of the System

In this section, the concept of system signature is used to define the Tsallis entropy of the remaining lifetime of a coherent system with an arbitrary system-level structure, assuming that all components of the system are functioning at time *t*. An *n*-dimensional vector $p=(p_{1},\ldots ,p_{n})$ whose *i*-th element $p_{i}=P\left(T=X_{i:n}\right),\;i=1,2,\ldots ,n;$ is the signature of such a system where $X_{i:n}$ is the *i*-th order statistic of the *n* independent and identically distributed (i.i.d.) component lifetimes $X=(X_{1},\ldots ,X_{n})$, that is, the time of the *i*-th component failure, and *T* is the failure time of the system; (see Samaniego [20]). Consider a coherent system with independent and identically distributed component lifetimes $X_{1},\ldots ,X_{n}$ and a known signature vector $p=(p_{1},\ldots ,p_{n})$. If $T_{t}^{1,n}=\left[T-t|X_{1:n}>t\right],$ represents the remaining lifetime of the system under the condition that at time t, all components of the system are functioning, then from the results of Khaledi and Shaked [21] the survival function of $T_{t}^{1,n}$ can be expressed as

(4) $$\begin{array}{ccc} P(T_{t}^{1,n}>x) & = & \sum_{i=1}^{n}p_{i}P\left(X_{i:n}-t>x|X_{1:n}>t\right),\\ & = & \sum_{i=1}^{n}p_{i}P\left({T_{t}}^{1,i,n}>x\right), \end{array}$$

where $T_{t}^{1,i,n}=\left[X_{i:n}-t|X_{1:n}>t\right],\;i=1,2,\cdots ,n,$ denotes the remaining lifetime of an *i*-out-of-*n* system under the condition that all components at time *t*. The survival and probability density functions of $T_{t}^{1,i,n}$ are given by

(5) P(Tt1,i,n>x)=∑k=0i−1nk1−St(x)kSt(x)n−k,x,t>0,

and

(6) $$f_{{T_{t}}^{1,i,n}}\left(x\right)=\frac{\Gamma (n+1)}{\Gamma (i)\Gamma (n-i+1)}{\left(1-S_{t}\left(x\right)\right)}^{i-1}{\left(S_{t}\left(x\right)\right)}^{n-i}f_{t}\left(x\right),\;x,t>0,$$

respectively, where Γ(·) is the complete gamma function. It follows that

(7) $$f_{T_{t}^{1,n}}\left(x\right)=\sum_{i=1}^{n}p_{i}f_{{T_{t}}^{1,i,n}}\left(x\right),\;x,t>0.$$

In what follows, we focus on the study of the Tsallis entropy of the random variable $T_{t}^{1,n}$, which measures the degree of uncertainty contained in the density of $[T-t|X_{1:n}>t],$ in terms of the predictability of the remaining lifetime of the system in terms of Tsallis entropy. The probability integral transformation $V=S_{t}\left(T_{t}^{1,n}\right)$ plays a crucial role in our goal. It is clear that $U_{i:n}=S_{t}\left(T_{t}^{1,i,n}\right)$ follows from a beta distribution with parameters n−i+1 and *i* with the PDF

(8) $$g_{i}\left(u\right)=\frac{\Gamma (n+1)}{\Gamma (i)\Gamma (n-i+1)}{\left(1-u\right)}^{i-1}u^{n-i},\;0<u<1,\;i=1,\cdots ,n.$$

In the forthcoming proposition, we provide an expression for the Tsallis entropy of ${T_{t}}^{1,n}$ by using the earlier transformation formulas.

**Theorem** **1.** *The Tsallis entropy of ${T_{t}}^{1,n}$ can be expressed as follows:*

(9) $$H_{\alpha }\left({T_{t}}^{1,n}\right)=\frac{1}{1-\alpha }\left[\int_{0}^{1}g_{V}^{\alpha }\left(u\right)f_{t}^{\alpha -1}\left(S_{t}^{-1}\left(u\right)\right)du-1\right],\;t>0,$$

*for all α>0.*

**Proof.**  By using the change of $u=S_{t}\left(x\right),$ from (2) and (6) we obtain

$$\begin{array}{ccc} H_{\alpha }\left({T_{t}}^{1,n}\right) & = & \frac{1}{1-\alpha }\left[\int_{0}^{\infty }{\left(f_{{T_{t}}^{1,n}}\left(x\right)\right)}^{\alpha }dx-1\right]\\ & = & \frac{1}{1-\alpha }\left[\int_{0}^{\infty }{\left(\sum_{i=1}^{n}p_{i}f_{{T_{t}}^{1,i,n}}\left(x\right)\right)}^{\alpha }dx-1\right]\\ & = & \frac{1}{1-\alpha }\left[\int_{0}^{1}{\left(\sum_{i=1}^{n}p_{i}g_{i}\left(u\right)\right)}^{\alpha }{\left(f_{t}\left(S_{t}^{-1}\left(u\right)\right)\right)}^{\alpha -1}dx-1\right]\\ & = & \frac{1}{1-\alpha }\left[\int_{0}^{1}g_{V}^{\alpha }\left(u\right){\left(f_{t}\left(S_{t}^{-1}\left(u\right)\right)\right)}^{\alpha -1}du-1\right]. \end{array}$$

In the last equality $g_{V}\left(u\right)=\sum_{i=1}^{n}p_{i}g_{i}\left(u\right)$ is the PDF of *V* denotes the lifetime of the system with independent and identically distributed uniform distribution. □

In the specail case, if we consider an *i*-out-of-*n* system with the system signature $p=(0,\ldots ,0,1_{i},0,\ldots ,0),\;i=1,2,\cdots ,n$, then Equation (9) reduces to

(10) $$H_{\alpha }\left(T_{t}^{1,i,n}\right)=\frac{1}{1-\alpha }\left[\int_{0}^{1}g_{i}^{\alpha }\left(u\right){\left(f_{t}\left(S_{t}^{-1}\left(u\right)\right)\right)}^{\alpha -1}du-1\right],$$

for all t>0.

The next theorem immediately follows by Theorem 1 from the aging properties of their components. We recall that *X* has increasing (decreasing) failure rate (IFR(DFR)) if $S_{t}\left(x\right)$ is decreasing (increasing) in *x* for all t>0.

**Theorem** **2.** *If X is IFR (DFR), then* $H_{\alpha }\left({T_{t}}^{1,n}\right)$ *is decreasing (increasing) in t for all* α>0*.*

**Proof.**  We just prove it when X is IFR where the proof for the DFR is similar. It is easy to see that $f_{t}\left(S_{t}^{-1}\left(u\right)\right)=u\lambda_{t}\left(S_{t}^{-1}\left(u\right)\right),\;0<u<1.$ This implies that Equation (9) can be rewritten as

(11) $$\begin{array}{c} \left(1-\alpha \right)H_{\alpha }\left({T_{t}}^{1,n}\right)+1=\int_{0}^{1}g_{V}^{\alpha }\left(u\right)u^{\alpha -1}{\left(\lambda_{t}\left(S_{t}^{-1}\left(u\right)\right)\right)}^{\alpha -1}du, \end{array}$$

for all α>0. On the other hand, one can conclude that $S_{t}^{-1}\left(u\right)=S^{-1}\left(uS\left(t\right)\right)-t,$ for all 0<u<1, and hence we have

(12) $$\lambda_{t}\left(S_{t}^{-1}\left(u\right)\right)=\lambda \left(S_{t}^{-1}\left(u\right)+t\right)=\lambda \left(S^{-1}\left(uS\left(t\right)\right)\right),\;0<u<1.$$

If $t_{1}\leq t_{2}$, then $S^{-1}\left(uS\left(t_{1}\right)\right)\leq S^{-1}\left(uS\left(t_{2}\right)\right)$. Thus, when *F* is IFR, then for all α>1(0<α≤1), we have

$$\begin{array}{ccc} \int_{0}^{1}g_{V}^{\alpha }\left(u\right)u^{\alpha -1}{\left(\lambda_{t_{1}}\left(S_{t_{1}}^{-1}\left(u\right)\right)\right)}^{\alpha -1}du & = & \int_{0}^{1}g_{V}^{\alpha }\left(u\right)u^{\alpha -1}{\left(\lambda (S^{-1}\left(uS\left(t_{1}\right)\right))\right)}^{\alpha -1}du\\ & \leq (\geq ) & \int_{0}^{1}g_{V}^{\alpha }\left(u\right)u^{\alpha -1}{\left(\lambda (S^{-1}\left(uS\left(t_{2}\right)\right))\right)}^{\alpha -1}du\\ & = & \int_{0}^{1}g_{V}^{\alpha }\left(u\right)u^{\alpha -1}{\left(\lambda_{t_{2}}\left(S_{t_{2}}^{-1}\left(u\right)\right)\right)}^{\alpha -1}du, \end{array}$$

for all $t_{1}\leq t_{2}.$ Using (11), we obtain

$$\begin{array}{c} \left(1-\alpha \right)H_{\alpha }\left({T_{t_{1}}}^{1,n}\right)+1\leq \left(\geq \right)\left(1-\alpha \right)H_{\alpha }\left({T_{t_{2}}}^{1,n}\right)+1, \end{array}$$

for all α>1(0<α≤1). This implies that $H_{\alpha }\left({T_{t_{1}}}^{1,n}\right)\geq H_{\alpha }\left({T_{t_{2}}}^{1,n}\right)$ for all α>0 and this completes the proof. □

The next example illustrates the results of Theorems 1 and 2.

**Example** **1.** *Consider a coherent system with system signature p=(0,1/2,1/4,1/4). The exact value of $H_{\alpha }\left({T_{t}}^{1,4}\right)$ can be calculated using the relation (9) given the lifetime distributions of the components. For this purpose, let us assume the following lifetime distributions.*

**Figure 1:**
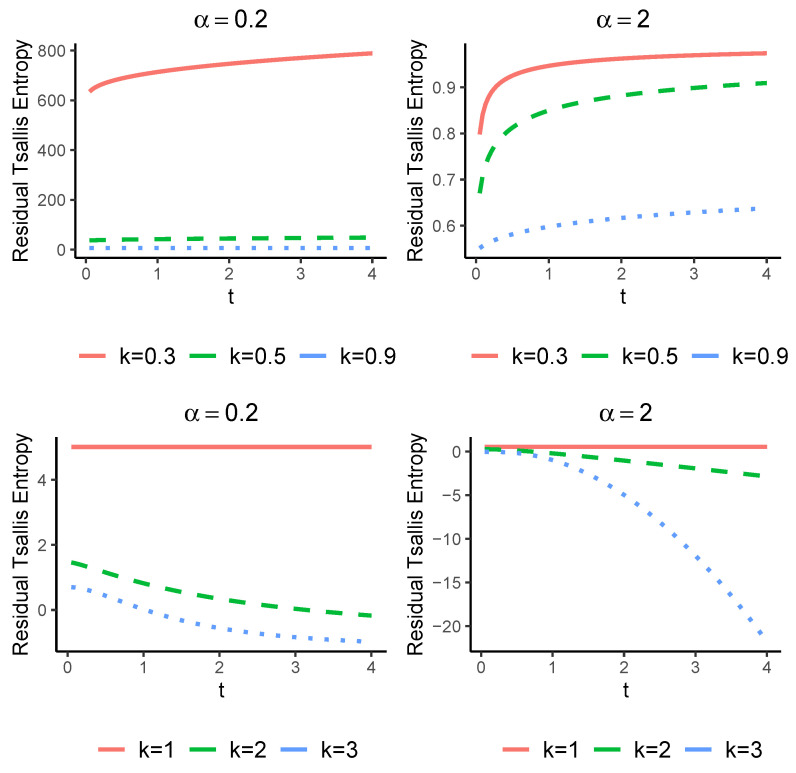
The exact values of $H_{\alpha }\left({T_{t}}^{1,4}\right)$ with respect to *t* for the Weibull distribution for values of α=0.2 and α=2 when k>0.

*(i)*  *Consider a Pareto type II with the survival function*
(13) $$S\left(t\right)={\left(1+t\right)}^{-k},\;k,t>0.$$
*It is not hard to see that*
$$H_{\alpha }\left({T_{t}}^{1,4}\right)=\frac{1}{1-\alpha }\left[{\left(\frac{k}{1+t}\right)}^{\alpha -1}\int_{0}^{1}u^{\frac{\left(\alpha -1)(k+1\right)}{k}}g_{V}^{\alpha }\left(u\right)du-1\right],\;t>0.$$
*It is obvious that the Tsallis entropy of $H_{\alpha }\left({T_{t}}^{1,4}\right)$ is an increasing function of time t. Thus, the uncertainty of the conditional lifetime ${T_{t}}^{1,4}$ increases as t increases. We recall that this distribution has the DFR property.*
*(ii)*  *Let us suppose that X has a Weibull distribution with the shape parameter k with the survival function*
(14) $$S\left(t\right)=e^{-t^{k}},\;k,t>0.$$
*After some manipulation, we have*
$$H_{\alpha }\left({T_{t}}^{1,4}\right)=\frac{1}{1-\alpha }\left[k^{\alpha -1}\int_{0}^{1}{\left(t^{k}-\log u\right)}^{\left(1-\frac{1}{k}\right)\left(\alpha -1\right)}u^{\alpha -1}g_{V}^{\alpha }\left(u\right)du-1\right],\;t>0.$$
*It is difficult to find an explicit expression for the above relation, and therefore we are forced to calculate it numerically. In Figure 1 we have plotted the entropy of ${T_{t}}^{1,4}$ as a function of time t for values of α=0,2 and α=2 and k>0. In this case, it is known that X is DFR when α=0,1. As expected from Theorem 2, it is obvious that $H_{\alpha }\left({T_{t}}^{1,4}\right)$ is increasing in t for α=0,1. The results are shown in Figure 1.*

Below, we compare the Tsallis entropies of two coherent systems from their lifetimes and their residual lifetimes.

**Theorem** **3.** *Consider a coherent system with independent and identically distributed IFR(DFR) component lifetimes. Then $H_{\alpha }\left({T_{t}}^{1,n}\right)\leq \left(\geq \right)H_{\alpha }\left(T\right)$ for all α>0.*

**Proof.**  We prove it when X is IFR where the proof for DFR property is similar. Since *X* is IFR, Theorem 3.B.25 of Shaked and Shanthikumar [19] implies that $X\geq_{d}X_{t},$ that is

$$f_{t}\left(S_{t}^{-1}\left(u\right)\right)\geq f\left(S^{-1}\left(u\right)\right),\;0<u<1,$$

for all t>0. If α>1(0<α<1), so we have

(15) $$\int_{0}^{1}g_{V}^{\alpha }\left(u\right)f_{t}^{\alpha -1}\left(S_{t}^{-1}\left(u\right)\right)du\geq \left(\leq \right)\int_{0}^{1}g_{V}^{\alpha }\left(u\right)f^{\alpha -1}\left(S^{-1}\left(u\right)\right)du,\;t>0.$$

Thus, from (9) and (15), we obtain

$$\begin{array}{ccc} H_{\alpha }\left({T_{t}}^{1,n}\right) & = & \frac{1}{1-\alpha }\left[\int_{0}^{1}g_{V}^{\alpha }\left(u\right)f_{t}^{\alpha -1}\left(S_{t}^{-1}\left(u\right)\right)du-1\right]\\ & \leq & \frac{1}{1-\alpha }\left[\int_{0}^{1}g_{V}^{\alpha }\left(u\right)f^{\alpha -1}\left(S^{-1}\left(u\right)\right)du-1\right]=H_{\alpha }\left(T\right). \end{array}$$

Therefore, the proof is completed. □

**Theorem** **4.** *If X is DFR, then a lower bound for $H_{\alpha }\left(T_{t}^{1,n}\right)$ is given as follows:*

$$H_{\alpha }\left({T_{t}}^{1,n}\right)\geq \frac{H_{\alpha }\left(T\right)}{S(t)}+\frac{1}{1-\alpha }\left(\frac{1}{S(t)}-1\right),$$

*for all α>0.*

**Proof.**  Since *X* is DFR, then it is NWU (i.e., $S_{t}\left(x\right)\geq S\left(x\right),\;x,t\geq 0.$) This implies that

$$S_{t}^{-1}\left(u\right)+t\geq S^{-1}\left(u\right),\;t\geq 0,$$

for all 0<u<1. On the other hand, it is known that when *X* is DFR, the PDF *f* is decreasing which implies that

$$f^{\alpha -1}\left(S_{t}^{-1}\left(u\right)+t\right)\leq \left(\geq \right)f^{\alpha -1}\left(S^{-1}\left(u\right)\right),\;0<u<1,$$

for all α>1(0<α<1). From (9), one can conclude that

$$\begin{array}{ccc} H_{\alpha }\left({T_{t}}^{1,n}\right) & = & \frac{1}{1-\alpha }\left[\int_{0}^{1}g_{V}^{\alpha }\left(u\right)\frac{f^{\alpha -1}\left(S_{t}^{-1}\left(u\right)+t\right)}{S(t)}du-1\right]\\ & \geq & \frac{1}{1-\alpha }\left[\int_{0}^{1}g_{V}^{\alpha }\left(u\right)\frac{f^{\alpha -1}\left(S^{-1}\left(u\right)\right)}{S(t)}du-1\right]\\ & = & \frac{1}{1-\alpha }\left[\frac{\left(1-\alpha \right)H_{\alpha }\left(T\right)+1}{S(t)}-1\right], \end{array}$$

for all α>0, and this completes the proof. □

## 3. Entropy Ordering of Two Coherent Systems

Given the imponderables of two coherent systems, this section discusses the partial ordering of their conditional lifetimes. Based on various existing orderings between the component lifetimes and their signature vectors, we find some results for the entropy ordering of two coherent systems. The next theorem compares the entropies of the residual lifetimes of two coherent systems.

**Theorem** **5.** *Let $T_{t}^{X,1,n}=\left[T-t|X_{1:n}>t\right]$ and $T_{t}^{Y,1,n}=\left[T-t|Y_{1:n}>t\right]$ denote the residual lifetimes of two coherent systems with the same signatures and n i.i.d component lifetimes $X_{1},\ldots ,X_{n}$ and $Y_{1},\ldots ,Y_{n}$ from cdfs F and G, respectively. If $X\;\leq_{d}Y$ and X or Y is IFR, then $H_{\alpha }\left(T_{t}^{X,1,n}\right)\leq H_{\alpha }\left(T_{t}^{Y,1,n}\right)$ for all α>0.*

**Proof.**  As a result of the relation (9), it is sufficient to demonstrate that $X_{t}\leq_{d}Y_{t}.$ Due to the assumption that $X\leq_{d}Y$ and *X* or *Y* is IFR, the proof of Theorem 5 of Ebrahimi and Kirmani [22] means that $X_{t}\leq_{d}Y_{t}$, and this concludes the proof. □

**Figure 2:**
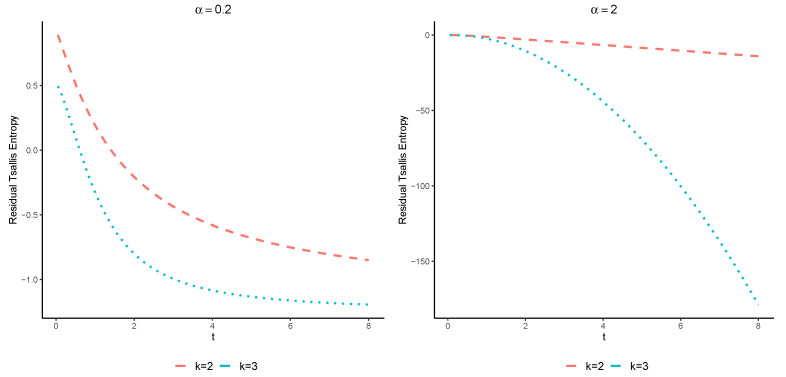
The exact values of $H_{\alpha }\left(T_{t}^{X,1,4}\right)$ (blue color) and $H_{\alpha }\left(T_{t}^{Y,1,4}\right)$ (red color) with respect to *t* for values of α=0.2 and α=2.

**Example** **2.** *Let us assume two coherent systems with residual lifetimes $T_{t}^{X,1,4}$ and $T_{t}^{Y,1,4}$ with the common signature $p=(\frac{1}{2},\frac{1}{4},\frac{1}{4},0).$ Suppose that X∼W(3,1) and Y∼W(2,1), where W(k,1) stands for the Weibull distribution with the survival function given in (14). It is easy to see that $X\leq_{d}Y.$ Moreover, X and Y are both IFR. Thus, Theorem 5 yields that $H_{\alpha }\left(T_{t}^{X,1,4}\right)\leq H_{\alpha }\left(T_{t}^{Y,1,4}\right)$ for all α>0. The plot of the Tsallis entropies of these systems is displayed in Figure 2.*

Next, we compare the residual Tsallis entropies of two coherent systems with the same component lifetimes and different structures.

**Theorem** **6.** *Let $T_{1,t}^{1,n}=\left[T_{1}-t|X_{1:n}>t\right]$ and $T_{2,t}^{1,n}=\left[T_{2}-t|X_{1:n}>t\right]$ represent the residual lifetimes of two coherent systems with signature vectors $p_{1}$ and $p_{2}$, respectively. Assume that the system’s components are independent and identically distributed according to the common CDF, F. Additionally, let $p_{1}\leq_{lr}p_{2}.$ Then,*

*(i)*  *if $f_{t}\left(S_{t}^{-1}\left(u\right)\right)$ is increasing in u for all t>0, then $H_{\alpha }\left(T_{1,t}^{1,n}\right)\geq H_{\alpha }\left(T_{2,t}^{1,n}\right)$ for all α>0.*
*(ii)*  *if $f_{t}\left(S_{t}^{-1}\left(u\right)\right)$ is decreasing in u for all t>0, then $H_{\alpha }\left(T_{1,t}^{1,n}\right)\leq H_{\alpha }\left(T_{2,t}^{1,n}\right)$ for all α>0.*

**Proof.**  (i) First, we note that the Equation (9) can be rewritten as follows:

(16) $$\left(1-\alpha \right)H_{\alpha }\left({T_{t_{i}}}^{1,n}\right)+1=\int_{0}^{1}g_{V_{i}}^{\alpha }\left(u\right)du\int_{0}^{1}g_{V_{i}}^{☆}\left(u\right){\left(f_{t}\left(S_{t}^{-1}\left(u\right)\right)\right)}^{\alpha -1}du,\left(i=1,2\right),$$

where $V^{☆}$ has the PDF as

$$g_{V}^{☆}\left(u\right)=\frac{g_{V}^{\alpha }\left(u\right)}{\int_{0}^{1}g_{V}^{\alpha }\left(u\right)du},\;0<u<1.$$

Assumption $s_{1}\leq_{lr}s_{2}$ implies $V_{1}\leq_{lr}V_{2}$, and this means that $V_{1}^{☆}\leq_{lr}V_{2}^{☆}$, which means that

$$\frac{g_{V_{2}}^{☆}\left(u\right)}{g_{V_{1}}^{☆}\left(u\right)}\propto {\left(\frac{g_{V_{2}}\left(u\right)}{g_{V_{1}}\left(u\right)}\right)}^{\alpha }$$

is increasing in *u* for all α>0, and hence, $V_{1}^{☆}\leq_{st}V_{2}^{☆}.$ When α>1(0<α<1), we obtain

(17) $$\begin{array}{ccc} \int_{0}^{1}g_{V_{1}}^{☆}\left(u\right){\left(f_{t}\left(S_{t}^{-1}\left(u\right)\right)\right)}^{\alpha -1}du & \leq (\geq ) & \int_{0}^{1}g_{V_{2}}^{☆}\left(u\right){\left(f_{t}\left(S_{t}^{-1}\left(u\right)\right)\right)}^{\alpha -1}du, \end{array}$$

where the inequality in (17) is obtained by noting that the conditions $V_{1}^{☆}\leq_{st}V_{2}^{☆}$ imply $E\left[\pi \left(V_{1}^{☆}\right)\right]\leq E\left[\pi \left(V_{2}^{☆}\right)\right]$ for all increasing (decreasing) functions π. Therefore, relation (16) gives

$$\left(1-\alpha \right)H_{\alpha }\left({T_{t_{1}}}^{1,n}\right)+1\leq \left(\geq \right)\left(1-\alpha \right)H_{\alpha }\left({T_{t_{2}}}^{1,n}\right)+1,$$

or equivalently, $H_{\alpha }\left({T_{1,t}}^{1,n}\right)\geq H_{\alpha }\left({T_{2,t}}^{1,n}\right)$ for all α>0. Part (ii) can be similarly obtained. □

The next example gives an application of Theorem 6.

**Figure 3:**
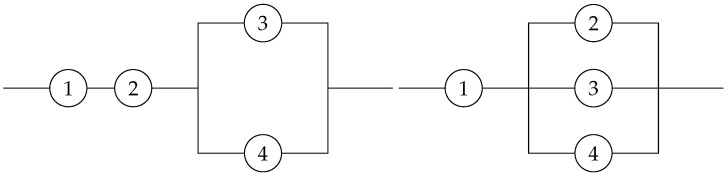
Two coherent systems with the likelihood ration ordered signature.

**Example** **3.** *Let us consider the two coherent systems of order 4 displayed in Figure 3 with residual lifetimes $T_{1,t}^{1,4}=\left[T_{1}-t|X_{1:4}>t\right]$ (left panel) and $T_{2,t}^{1,4}=\left[T_{2}-t|X_{1:4}>t\right]$ (right panel). It is not hard to see that the signatures of these systems are $p_{1}=\left(\frac{1}{2},\frac{1}{2},0,0\right)$ and $p_{2}=\left(\frac{1}{4},\frac{1}{4},\frac{1}{2},0\right),$ respectively. Assume that the component lifetimes are independent and identically distributed according to the following survival function,*

$$S\left(t\right)={\left(1+t\right)}^{-2},\;t>0.$$

*After some calculation, one can obtain $f_{t}\left(S_{t}^{-1}\left(u\right)\right)=\frac{2u\sqrt{u}}{1+t},\;t>0.$ This function is increasing in u for all t>0. Hence, due to Theorem 6, it holds that $H_{\alpha }\left(T_{1,t}^{1,4}\right)\geq H_{\alpha }\left(T_{2,t}^{1,4}\right)$ for all α>0.*

## 4. Some Useful Bounds

When the complexity is high and the number of components is large, it is difficult to compute the $H_{\alpha }\left(T_{t}^{1,n}\right)$ of a coherent system. This situation is frequently encountered in practice. Under such circumstances, a Tsallis entropy bound can be useful to estimate the lifetime of a coherent system. To see some recent research on bounds on the uncertainty of the lifetime of coherent systems, we refer the reader, for example, to Refs. [15,16,23] and the references there. In the following theorem, we provide bounds on the residual Tsallis entropy of the lifetime of the coherent system in terms of the residual Tsallis entropy of the parent distribution $H_{\alpha }\left(X_{t}\right)$.

**Theorem** **7.** *Let $T_{t}^{1,n}=\left[T-t|X_{1:n}>t\right]$ represent the residual lifetime of a coherent system consisting of n independent and identically distributed component lifetimes having the common CDF F with the signature $p=(p_{1},\cdots ,p_{n})$. Suppose that $H_{\alpha }\left(T_{t}^{1,n}\right)<\infty$ for all α>0. It holds that*

(18) $$H_{\alpha }\left(T_{t}^{1,n}\right)\geq {\left(B_{n}\left(p\right)\right)}^{\alpha }H_{\alpha }\left(X_{t}\right)+\frac{{\left(B_{n}\left(p\right)\right)}^{\alpha }-1}{1-\alpha },$$

*for all α>1 and*

(19) $$H_{\alpha }\left(T_{t}^{1,n}\right)\leq {\left(B_{n}\left(p\right)\right)}^{\alpha }H_{\alpha }\left(X_{t}\right)+\frac{{\left(B_{n}\left(p\right)\right)}^{\alpha }-1}{1-\alpha },$$

*for 0<α<1 where $B_{n}\left(p\right)=\sum_{i=1}^{n}p_{i}g_{i}\left(p_{i}\right),$ and $p_{i}=\frac{n-i}{n-1}.$*

**Proof.**  It can be clearly verified that the mode of the beta distribution with parameters n−i+1 and *i* is $p_{i}=\frac{n-i}{n-1}.$ Therefore, we obtain

$$g_{V}\left(v\right)\leq \sum_{i=1}^{n}p_{i}g_{i}\left(p_{i}\right)=B_{n}\left(p\right),\;0<v<1.$$

Thus, for α>1(0<α<1), we have

$$\begin{array}{ccc} 1+\left(1-\alpha \right)H_{\alpha }\left(T_{t}^{1,n}\right) & = & \int_{0}^{1}g_{V}^{\alpha }\left(v\right)f_{t}^{\alpha -1}\left(S_{t}^{-1}\left(v\right)\right)dv\\ & & \leq {\left(B_{n}\left(p\right)\right)}^{\alpha }\int_{0}^{1}f_{t}^{\alpha -1}\left(S_{t}^{-1}\left(v\right)\right)dv\\ & = & {\left(B_{n}\left(p\right)\right)}^{\alpha }\left[\left(1-\alpha \right)H_{\alpha }\left(X_{t}\right)+1\right]. \end{array}$$

The last equality is obtained from (3), from which the desired result follows. □

The bounds given in (18) and (19) are very valuable when the number of components is large or the structure of the system is complicated. Now, we obtain a public lower bound using properties of the Tsallis information measure and mathematical concepts.

**Theorem** **8.** *Under the requirements of the Theorem 7, we have*

(20) $$H_{\alpha }\left(T_{t}^{1,n}\right)\geq H_{\alpha }^{L}\left(T_{t}^{1,n}\right),$$

*where $H_{\alpha }^{L}\left(T_{t}^{1,n}\right)=\sum_{i=1}^{n}p_{i}H_{\alpha }\left({T_{t}}^{1,i,n}\right)$ for all α>0.*

**Proof.**  Recalling Jensen’s inequality for the convex function $t^{\alpha }$ (it is concave (convex) for 0<α<1(α>1)), it holds that

$${\left(\sum_{i=1}^{n}p_{i}f_{T_{t}^{1,i,n}}\left(x\right)\right)}^{\alpha }\geq \;\left(\leq \right)\sum_{i=1}^{n}p_{i}f_{T_{t}^{1,i,n}}^{\alpha }\left(x\right),\;t>0,$$

and hence, we obtain

(21) $$\left(\int_{0}^{\infty }f_{T_{t}^{1,n}}^{\alpha }\left(x\right)dx\right)\geq \;\left(\leq \right)\left(\sum_{i=1}^{n}p_{i}\int_{0}^{\infty }f_{T_{t}^{1,i,n}}^{\alpha }\left(x\right)dx\right).$$

Since 1−α>0(1−α<0), by multiplying both sides of (21) in 1/(1−α), we obtain

$$\begin{array}{ccc} H_{\alpha }\left(T\right) & \geq & \frac{1}{1-\alpha }\left[\sum_{i=1}^{n}p_{i}\int_{0}^{\infty }f_{T_{t}^{1,i,n}}^{\alpha }\left(x\right)dx-1\right]\\ & = & \frac{1}{1-\alpha }\left[\sum_{i=1}^{n}p_{i}\int_{0}^{\infty }f_{T_{t}^{1,i,n}}^{\alpha }\left(x\right)dx-\sum_{i=1}^{n}p_{i}\right]\\ & = & \sum_{i=1}^{n}p_{i}\left[\frac{1}{1-\alpha }\left(\int_{0}^{\infty }f_{T_{t}^{1,i,n}}^{\alpha }\left(x\right)dx-1\right)\right]\\ & = & \sum_{i=1}^{n}p_{i}H_{\alpha }\left({T_{t}}^{1,i,n}\right), \end{array}$$

and this completes the proof. □

Notice that the equality in (20) holds for *i*-out-of-*n* systems in the sense that we have $p_{j}=0$, for j≠i, and $p_{j}=1$, for j=i, and then $H_{\alpha }\left(T_{t}^{1,n}\right)=H_{\alpha }\left(T_{t}^{1,i,n}\right)$. When the lower bounds for 0<α<1 in both parts of Theorems 7 and 8 can be computed, one may use the maximum of the two lower bounds.

**Figure 4:**
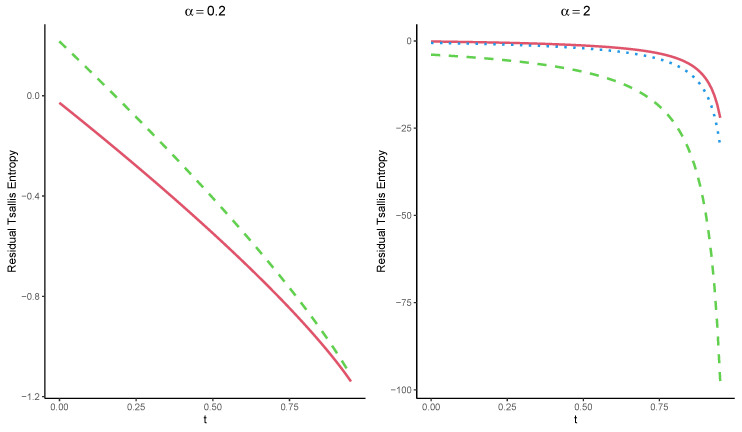
Exact value of $H_{\alpha }\left(T_{t}^{1,3}\right)$ (solid line), as well as the corresponding lower bounds (18) (dashed line) and (19) (dotted line) for the standard uniform distribution concerning time t.

**Example** **4.** Let $T_{t}^{1,5}=\left[T-t|X_{1:5}>t\right]$ represent the residual lifetime of a coherent system with the signature $p=(0,\frac{3}{10},\frac{5}{10},\frac{2}{10},0)$ consisting of n=5 independent and identically distributed component lifetimes having a uniform distribution in [0,1]. It is easy to verify that $B_{5}\left(p\right)=2.22.$ Thus, by Theorem 7, the Tsallis entropy of $T_{t}^{1,5}$ is bounded for α>1(0<α<1), as follows:

$$H_{\alpha }\left(T_{t}^{1,n}\right)\geq \frac{2.22^{\alpha }{\left(1-t\right)}^{1-\alpha }-1}{1-\alpha },$$

for all α>1 and

$$H_{\alpha }\left(T_{t}^{1,n}\right)\leq \frac{2.22^{\alpha }{\left(1-t\right)}^{1-\alpha }-1}{1-\alpha },$$

for 0<α<1. Moreover, the lower bound given in (20) can be obtained as follows:

(22) $$H_{\alpha }\left(T_{t}^{1,3}\right)\geq \frac{1}{1-\alpha }\left[{\left(1-t\right)}^{1-\alpha }\sum_{i=1}^{n}p_{i}\int_{0}^{1}g_{i}^{\alpha }\left(u\right)du-1\right],\;t>0,$$

for all α>0. Assuming uniform distribution for the component lifetimes, we computed the bounds given by (19) (dashed line), as well as the exact value of $H_{\alpha }\left(T_{t}^{1,3}\right)$ obtained directly from (9), and also the bounds given by (22) (dotted line). The results are displayed in Figure 4. As we can see, regarding the lower bound in (22) (dotted line) for α>1, it is better than the lower bound given by (19).

## 5. Conclusions

Intuitively, it is better to have systems that work longer and whose remaining life is less uncertain. We can make more accurate predictions when a system has low uncertainty. The Tsallis entropy of a system is an important measure for designing systems based on these facts. If we have some information about the lifetime of the system at time *t*, for example, that the system will still function at age *t*, then we may be interested in quantifying the predictability of the remaining lifetime. In this work, we presented a simple assertion for the Tsallis entropy of the system lifetime for the case where all components contained in the system are in operation at time *t*. Several properties of the proposed measure were discussed. In addition, some partial stochastic orderings between the remaining lifetimes of two coherent systems were discussed in terms of their Tsallis entropy using the concept of a system signature. Numerous examples were also given to illustrate the results.

## Data Availability

No new data were created or analyzed in this study. Data sharing is not applicable to this article.
